# Medical students’ creative projects on a third year pediatrics clerkship: a qualitative analysis of patient-centeredness and emotional connection

**DOI:** 10.1186/s12909-016-0614-4

**Published:** 2016-03-16

**Authors:** Johanna Shapiro, Diane Ortiz, You Ye Ree, Minha Sarwar

**Affiliations:** Department of Family Medicine, University of California Irvine School of Medicine, 101 City Dr. South, Bldg 200, Rte 81, Ste 835, Orange, CA 92868 USA; University of California Irvine, Irvine, USA; Touro University School of Osteopathic Medicine, Henderson, NV USA

**Keywords:** Pediatrics clerkship, Student-patient relationship, Reflection, Medical humanities

## Abstract

**Background:**

Increasingly, medical educators are incorporating reflective writing and original creative work into educational practices with the goals of stimulating student self-awareness, appreciation of multiple perspectives, and comfort with ambiguity and uncertainty. This study investigated students’ creative projects to assess the extent to which they adopted a patient/relationship-centered, emotionally connected position toward patients and families.

**Methods:**

Over a 10 year period, students on a required third year pediatrics clerkship individually or in groups completed either a reflection or an education project using a creative medium. 520 projects (representing 595 students, 74.7 % of total eligible students) were qualitatively analyzed according to various thematic and emotion-based dimensions.

**Results:**

The majority of projects were personal narrative essays and poetry. The largest number of project *themes* related to the importance of patient/relationship-centered medicine with patients. The next largest number of projects focused on health education of parents, patients, or classmates. In telling their stories, students were more likely to use a personal *voice* representing either their or the patient’s perspective than an objective, impersonal one. In terms of *emotional tone*, projects were more likely to be serious than humorous. The largest number of students’ emotions expressed an empathic tone. Students identified a large number and wide range of both negative and positive feelings in themselves and their patients. The majority of student emotions were positive, while the majority of patient and family emotions were negative.

**Conclusions:**

Students’ preference for patient-centered, relational themes, as well as their tendency to favor the first voice, empathic tone, and willingness to express a range of positive and negative emotions in presenting their projects, suggests that they valued emotional connection with patients and families during the pediatrics clerkship experience.

## Background

Although pediatric clerkships are guided by goals and objectives specified by the APA/COMSEP General Pediatric Clerkship Curriculum [1], we know little about the subjective experiences of students in this required clinical training. While achievement of objective goals is assessed through various methods [2, 3], we lack effective ways of examining students’ thoughts and feelings about clerkship participation. Yet we know that students seek from their Pediatrics clerkship not only scientifically rigorous instruction, but also other kinds of humanistic learning [4].

In recent years, medical education has evinced a growing interest in incorporating reflective practices into medical student and resident training. Reflection is implicated in core ACGME competencies [5], and reflective writing is considered to be a means of deepening empathy [6], enhancing learner wellbeing, stimulating critical thinking [7], developing ethical mindfulness [8], stimulating tolerance, and enhancing capacity to manage complexity and ambiguity [9]. A systematic review of reflective writing concluded that the practice could make a contribution to improving empathy in medical students [10]. Reflection activities most often take the form of written exercises, but may include the use of other creative media [11].

Reflective practices occasionally have been incorporated into pediatrics training. In one such report [12], the purpose of student reflections was to identify factors that encouraged/discouraged connection with patients and families. Thematic analysis identified limitations of time, knowledge, language and culture. A summary of scholarly literature examining the use of reflective writing and creative projects in preclinical and clinical medical education suggests that students used such assignments to examine professional development, explore relational issues, retain humanistic attitudes, understand the patient’s experience, deal with personal fallibility, and cope with stress [13–18].

Of particular interest in examining the products of reflective activities in medical education is their ability to provide insight into learner and patient emotions. Research on doctor-patient communication has long noted the role emotional factors play in clinical encounters because of their relationship to adherence, compliance, and satisfaction in patients [19, 20]. Yet medical school curricula pay insufficient attention to educating learners about patients’ emotions or their own [21, 22]. Medical students are often embarrassed and uncomfortable when confronted with patients’ emotions [23, 24]. In one study, students identified patients who expressed negative emotions as making patient-centered care more difficult [25]. In simulated settings, the unpredictability of standardized patients’ emotions can be unsettling to learners [26], although students are also able to respond to standardized patients’ emotional cues [27].

Medical students struggle with their own feelings over the course of training [28–30]. Emotional disequilibrium is an important contributor to overall medical student distress [31]. One study found that, in student essays, negative emotion words increased from the pre-clinical to the clinical years [32]. As medical education progresses, students appear to become less able to regulate their emotions, less able to express empathic concern, and more distressed by the emotions of others [33]. Another study found many inhibitors to empathy in the hidden curriculum, including ideals of detachment and objectivity, and noted that students use cynicism as a coping strategy [34]. However, a recent study based on reflective letter-writing to patients by graduating 4^th^ year medical students found more emotional awareness [35].

The present study examined required student creative projects, including both written and artistic efforts, to understand how students represent their experiences, particularly their own emotions and those of patients, families, and physicians, as part of a third year pediatrics clerkship. By analyzing the thoughts and feelings of medical students, our study provides new knowledge about the socialization experience they undergo during this aspect of their medical education. Considering students’ creative efforts as a form of data can provide insight into tacit knowledge and deep learning [36].

## Methods

The third year Pediatrics clerkship at this mid-sized American public university school of medicine is a required 8 week (over the course of the study period, reduced to 6 week) rotation. Groups of approximately 15–20 students rotate through the pediatric inpatient service, newborn nursery, pediatric ambulatory settings, and subspecialty clinics. The patient population is varied, but includes a large number of low-income, Hispanic families.

As part of the clerkship, students are required to complete either a “reflection” project or a patient/parent/peer health education project using a creative medium. Instructions for the reflection project are provided at the start of the clerkship, and ask students to describe or portray a memorable patient/family encounter, lessons learned from the clerkship, an ethical dilemma, or thoughts about pediatrics in general. Instructions for the education project ask students to provide information for either parents, patients, or their classmates about a pediatric medical problem (such as juvenile diabetes) or a public health issue (such as drowning or second-hand smoke). The instructions for both reflection and education projects were developed by the first author and the clerkship director and remained the same throughout the study period. Students could work individually or in groups of 4 or 5. The projects were due during the final week of the clerkship so give students the opportunity to have as much exposure to Pediatrics as possible.

Since evidence exists that reflective activities alone are not as effective as reflection combined with group discussion in terms of problem analysis, identifying different perspectives, and exploring solutions [37], we used a moderately large group discussion (*n* = 15–18), facilitated by the clerkship director and the first author (a psychologist), to further explore the meaning of student projects. Projects were not graded; however, students received individualized written feedback from the first author based on the formative rubric developed by Wald et al. [38], a practice widely used to extend the value of the initial reflection and solidify learning [5].

### Data analysis

Student projects were identified as the unit of analysis. The projects were first reviewed by a research team consisting of the first author and several medical students. Using a grounded theory approach [39, 40], each reviewer independently studied a subset of the projects 2–3 times, “deconstructing” both individual themes and emotions expressed in the projects. The research team compared their initial observations in an iterative process that involved both ongoing coding and identification of new codes as needed. Projects coded earlier were reviewed to ensure inclusion of more recently discovered categories. We grouped the codes used in scoring each project into broader categories addressing the theme(s) and emotions expressed in each project. The research team members met as a group and by email to discuss category issues, refine the scoring options, and resolve differences. Using this method, numerical counts and frequency distributions were computed for all categories and subcategories.

Codes included year project was completed, gender of student(s) completing project, type of project (artistic medium employed), and point of view represented (medical student’s, patient’s, family member’s, or resident/attendings’). In our analysis of type of project, we examined poetry and prose separately, in case different types of writing emphasized different themes and emotions. We also identified theme and emotion-based codes. Themes referred to the main point, focus, or message of the project. Emotional expression included *all* the emotions expressed in the project. Each project could be coded multiple times to ensure that all themes, emotions, and perspectives were captured. Forty-two theme codes and 31 emotion codes were identified.

We also considered regroupings of related content themes, for example combing various codes into an umbrella grouping that we re-labeled **patient/relationship-centered care**. We also summarized emotional *tone* into categories of positive (13 codes), reflective (3 codes), and negative (15 codes). These determinations were based on face validity. We further conducted analyses examining the interaction of themes and emotions. To facilitate interpretation of these complex relationships, we calculated the average number of emotions per theme so that we could determine unusually high or low frequencies of emotions. As well, we coded for shifts in emotions and attitudes within a given project. Finally we considered the data by year of project (collapsed into three approximately equal groups: Group 1, 2002–2005, *N* = 181; Group 2, 2006–2008, *N* = 156; Group 3, 2009–2013, *N* = 185) and gender of student to identify differences or patterns.

This study was reviewed by the University of California Irvine Institutional Review Board, which granted expedited retrospective approval (HS 2013–9691). The study was also approved by a Family Educational Rights and Privacy Act (FERPA) analyst to ensure that students’ privacy and confidentiality would not be violated. Because of the retrospective nature of the study and because all data were completely de-identified prior to data analysis, IRB and FERPA did not require a formal consent process. This research was conducted in conformity to the World Medical Association Declaration of Helsinki.

## Results

### Study period and sample

**Fig. 1:**
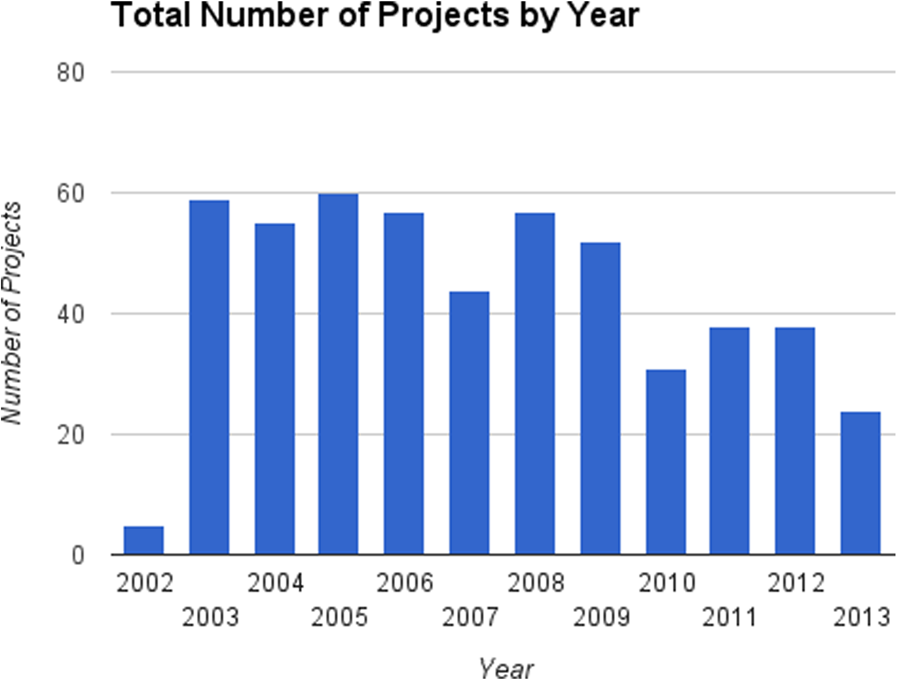
Total projects by year

Data were collected from July 2003 to Jun 2013. Each year, due to scheduling conflicts, 1–2 clerkship rotations were unable to offer the creative project reflection session. Over the 10 year period of the study, 797 students participated in the clerkship and 595 (74.7 %) submitted usable projects. Of the students submitting projects, 51.8 % were males. Usable projects per year ranged from 24 to 65 (Fig. 1). The total number of projects submitted was 520 (some students collaborated on a single project).

### Types of projects

**Fig. 2:**
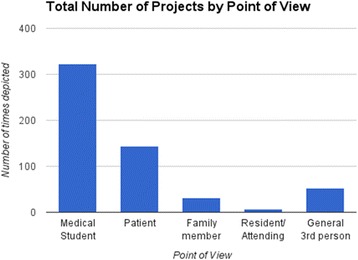
Total projects by point of view

In terms of type of projects (Fig. 2), 78.1%were written (36.9 % of written projects were poetry), 26.4 % were arts-based, and 12.5 % were health education. The majority of written projects were stories about the student’s encounter with a patient, followed closely by poetry and personal essays. Despite the predominance of written approaches, there was great variety in the projects, including sketches, drawings, paintings, collage, sculptures, posters, skits, games, video presentations, and songs. Over time, health education projects increased from Group 1 to Groups 2 and 3 (12.3 %/35.4 %/52.3 %).

### Point of view

**Fig. 3:**
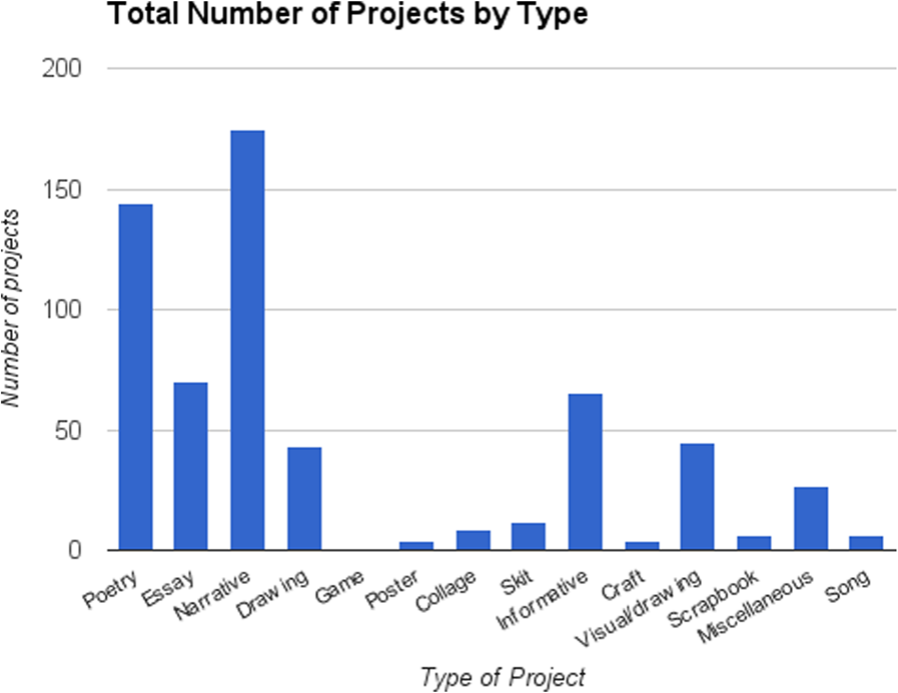
Total projects by type

The large majority of projects adopted the perspective of the medical student, followed by that of the patient (Fig. 3). Students sometimes adopted multiple points of view in a single project. The number of students using the third person point of view increased in Group 3 (55.8 %), compared to Groups 1 (26.9 %) and 2 (17.3 %). Regarding type of project, prose projects adopted the medical student’s perspective (70.4 %) slightly more than did arts projects (61.2 %), followed by education projects (58.5 %) and poetry (53.3 %). Conversely, poetry projects tended to adopt the patient’s perspective (40.7 %) much more often than any other type of project (23.3 % prose; 23.0 % art; education, too few to calculate). An objective, third person point of view was used in over one-third of education projects (36.9 %) but rarely in other kinds of projects (14.4 % art; 5.3 % poetry; 3.5 % prose).

### Themes

**Fig. 4:**
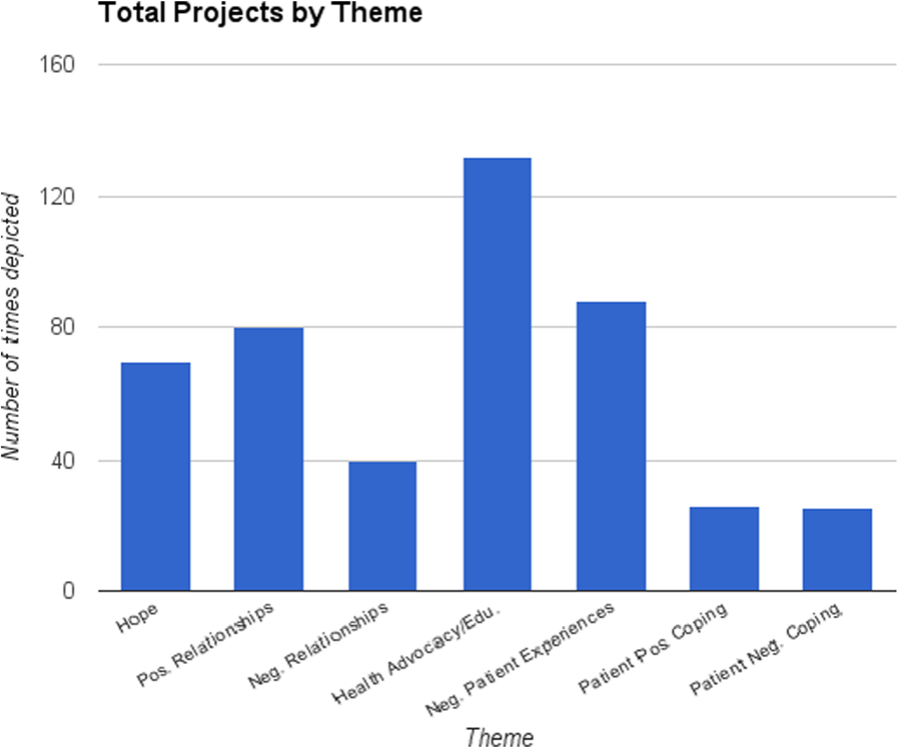
Total projects by theme

We identified a total of 42 themes, with a total of 831 thematic references (Fig. 4). The largest number - was related to patient/relationship-centered care (22.0 %). The next largest number addressed health education (15.9 %). Other thematic references endorsed the importance of hope (8.4 %); and identification with either family or child (6.7 %). Smaller numbers addressed a wide range of topics.

Regarding differences by year for themes, Group1 tended to comment more often on negative relationships with patients and family members (45.0 %) than did Groups 2 and 3 (25.0 %/30.0 %). Themes depicting negative patient experiences decreased from Group 1 to Groups 2 and 3 (43.2 %/26.1 %/30.7 %). Negative patient coping was less commented on in Groups 2 and 3 compared to Group 1 (24.0 %/28.0 %/48.0 %). The theme of student stress decreased markedly from Group 1 to Groups 2 and 3 (65.2 %/21.7 %/13.1 %). In Groups 2 and 3 educational themes increased compared to Group 1 (37.1 %/44.7 %/18.2 %).

In terms of thematic differences in different types of projects, education projects expressed almost never referenced themes other than health education. Art projects also frequently incorporated health education themes (48.9 %) compared to prose and poetry (10.1 %/7.3 %). Arts projects expressed far fewer themes than written projects. Arts and education projects expressed hope (12.7 %/10.1 %) only about half as often as did prose projects (24.4 %). In terms of negative relationships, art projects noted these least often (12.9 %), and prose and poetry more often (15.6 %/20.0 %).

### Overall emotional tone

**Fig. 5:**
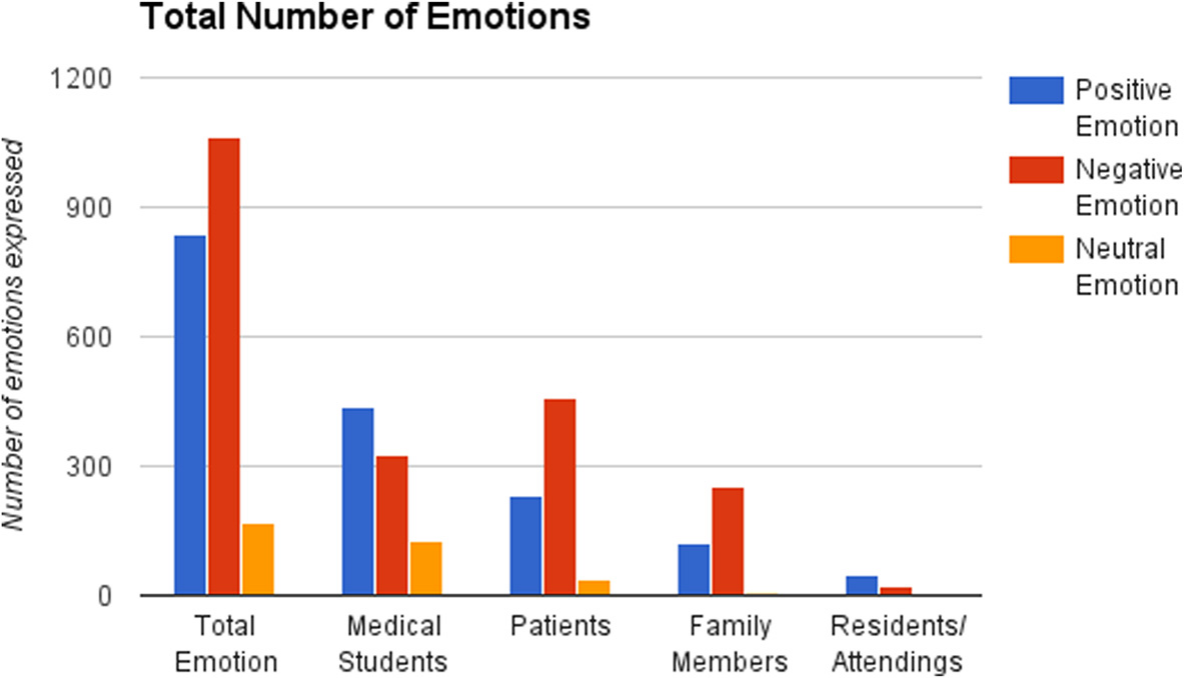
Total number of emotions

Across all projects, students made 2063 emotional references. Of these, 51.5 % were negative emotions, 40.5 % were positive emotions, and 8.0 % were reflective attitudes. The overwhelming majority of the projects used a serious tone (only7.5 % of projects were coded as primarily humorous). The most frequently expressed emotions were the cluster of worry/concern/anxiety. Projects were also likely to express empathy, optimism/hope, sadness, fear, frustration, and pain (Fig. 5). Compared to prose (5.4 emotions/project) and poetry (4.2 emotions/project), arts (3.6 emotions/projects) and education (2.6 emotions/project) projects expressed relatively few emotions.

### Student emotions

Projects represented student emotions more frequently than any other group (43.0 %). The largest number of emotions describing students was positive (49.0 %), followed by negative (37.0 %), and reflective (14.0 %). Students frequently described themselves as empathic and understanding of the patient’s perspective (25.5 % of total student positive emotions). Students also reported feeling optimistic and hopeful (16.6 %), and saw themselves as friendly (16.6 %), as well as feeling respect/admiration for patients and family (8.7 %). The negative emotion students expressed most frequently was worry/anxiety for patient and family (41.5 % of total student negative emotions). Other common negative emotions students noted were feelings of frustration (12.5 %) and sadness (9.5 %). Although student worry/anxiety remained about the same across all year-groups (14.0 %/15.0 %/15.6 %), Group 1 reported somewhat more negative student emotions such as frustration, confusion, distress, pessimism, and guilt than did Groups 2 and 3 (41.5 %/34.3 %/34.7 %). Positive student emotions peaked in Group 2 (56.7 %) compared to 44.8 % in Group 1 and 48.1 % in Group 3. Empathy/understanding decreased from equal percentages in Groups 1 and 2 (14.8 %/14.6 %) to only 8.2 % in Group 3, while feelings of friendliness increased from Group 1 to Groups 2 and 3 (4.2 %/9.4 %/11.7 %). Prose projects expressed many more positive student emotions than did poetry, art, or education projects (101.2 %/78 %/70.5 %/60.0 %).

### Patient emotions

The total number of patient emotions represented in projects was somewhat less than student emotions, but still a large number (35.1 % of total emotions). In contrast to students, patients were portrayed as having negative emotions about twice as often as positive emotions (67.2 % vs. 31.8 %). In exploring patients’ negative emotions, the most common feeling identified was fear (18.5 % of all negative patient emotions), followed closely by pain/distress (15.8 %). Other common negative patient emotions were worry/anxiety (11.1 %), sadness/melancholy (10.7 %) and frustration (9.9 %). Some projects showed patient happiness (18.7 % of all positive patient emotions), contentment (14.3 %), optimism/hope (12.6 %), and relief (11.7 %). Across year-groups, there were no striking shifts in percentages of negative emotions (66.1 %/60.5 %/64.1 %); but there was an increase in positive emotions from Group 1 to Groups 2 and 3 (28.7 %/33.3 %/34.6 %). Education projects also expressed far fewer than other projects, followed by art, poetry, and prose (29.2 %/64.7 %/82.0 %/91.1 %).

### Family emotions

Only 381 emotions (18.5 % of total emotions) were represented for family members. Negative emotions appeared about twice as often as positive emotions (66.9 % vs.31.8 %). Projects that addressed family feelings portrayed worry/anxiety most often (29.8 %). Less often, love (28.1 %) and sadness (14.1 %) emerged in projects, with slightly smaller numbers showing distress (9.4 %), optimism/hope (17.4 %), frustration (7.8 %), confusion (7.5 %), and anger (7.1 %). There were no major differences across years for references to either positive or negative family emotions. Prose projects expressed many more negative family emotions (67.3 %) than did poetry, art, or education projects (34.7 %/28.1 %/13.8 %). Education and art projects represented somewhat fewer positive family emotions (13.8 %/22.3 %) compared to prose or poetry projects (28.0 %/34.7 %).

### Resident/attending emotions

Emotions of residents and attending physicians were rarely identified (only 3.5 % of total emotions), regardless of type of project. More positive than negative emotions were noted (68.1 % vs.30.6 %), with “friendliness” being the most common (32.7 %). Equal but smaller numbers showed either empathy/understanding or anger/judgmentalness in physician superiors Negative physician emotions decreased creased noticeably from Groups 1 to 2 and 3 (41.7 %/26.7 %/4.3 %); while positive physician emotions increased (55.6 %/73.3 %/85.7 %).

Overall, in their creative projects students expressed a wide range of both negative and positive feelings regarding themselves, patients, and family members..For medical students and residents/attendings, more positive than negative emotions were identified. For patients and families, the reverse was true. There were more negative emotions in Group 1 than in the subsequent two groups. There were notably fewer emotions expressed in health and arts projects than in written projects.

### Interaction of themes and emotions: medical student emotions

**Fig. 6:**
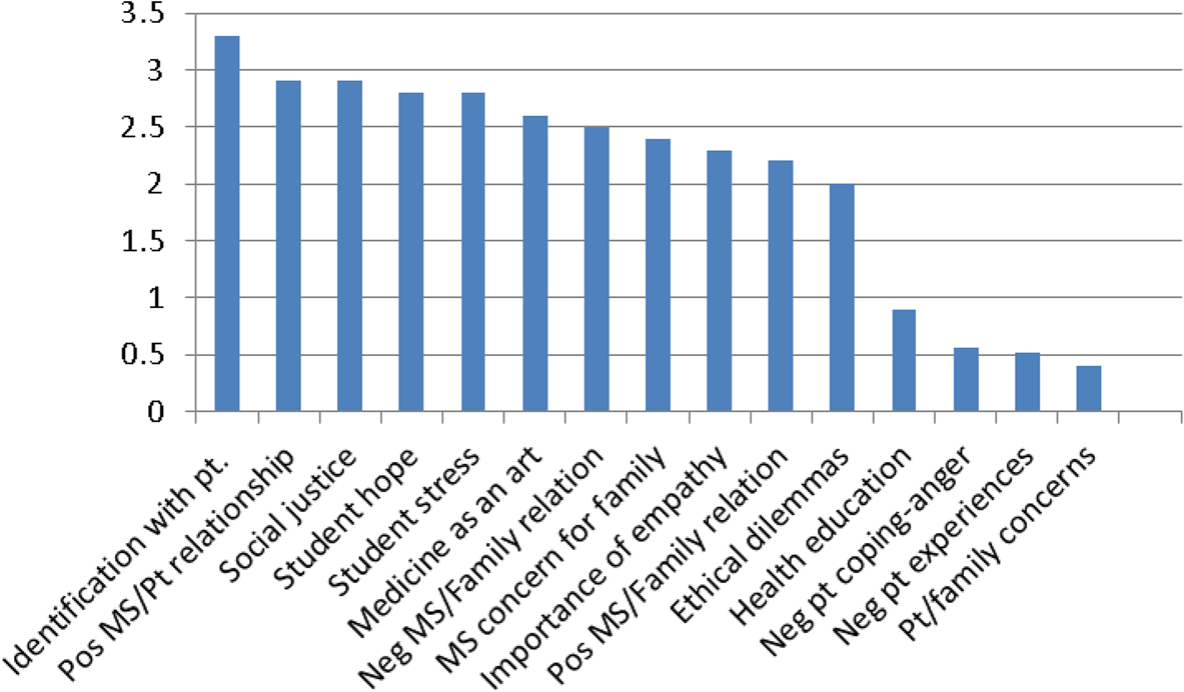
Medical students: examples of high and low number of emotions by theme

The average number of student emotions expressed per theme was about 1.5. In the largest thematic category (patient/relationship-centered medicine), students expressed a higher-than-average number of personal emotions (2.3). Educational projects showed a below-average ratio of student emotions to theme (0.9). Student projects tended to express the most emotions when students were strongly identified with the patient; they had a positive relationship with the patient; they were hopeful and optimistic; or they were concerned about issues of social justice or their own stress. When projects focused on negative themes, such as patient and family concerns, negative patient experiences; and angry negative patient coping, students used a smaller number of emotional words in describing themselves (Fig. 6 shows examples of themes with either high or low numbers of student emotions).

### Interaction of themes and emotions: patient emotions

**Fig. 7:**
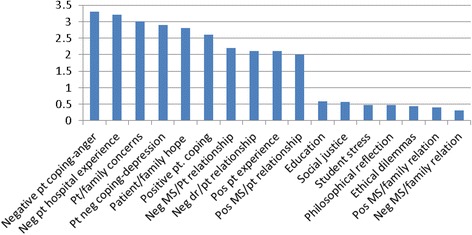
Patients: examples of high and low number of emotions by theme

The average number of patient emotions per theme was 1.7. The overall thematic category of patient-/relationship-centered medicine generated a slightly lower than average number of patient emotions (1.1). Education themes generated an even lower number (0.59). Themes that expressed the highest number of patient emotions were negative ones: patient anger, acting out, or withdrawal; negative hospital experiences; patient/family concerns for the future; patient withdrawal and depression; negative student- and doctor-patient relationships. Certain positive themes were associated with higher emotions as well: patient and family hope; positive patient coping, and positive medical student-patient relationships. The themes that generated the least emotions were ones in which the patient was not directly involved (Fig. 7 shows examples of high and low frequency of patient emotions by theme).

### Interaction of themes and emotions: family members

The average number of emotions represented per project for family members was 0.98. Unsurprisingly, themes associated with family members themselves recorded the most number of emotions: student’s identification with a family member; negative family experiences in the hospital; family hope and so forth. The theme of family members’ positive relationship with students had 2.1 emotions. Themes not directly related to families reported few family emotions. Overall, patient/relationship-centered themes generated a lower than average ratio of parent emotions (0.84) and health education projects also generated a very low number (0.30).

Emotions expressed for almost all themes were both negative and positive, although skewing toward the overall valence of the theme (e.g., the ratio of negative to positive emotions was 14:8 for the theme “negative student-patient relationships”). Sometimes the same theme elicited either a positive or a negative emotion from different students; and occasionally the same theme elicited both positive and negative emotions from a single student.

### Shifts in emotions and attitudes

Only 170 total shifts were identified. We found two major “changes.” The largest number of shifts was from a negative to a positive attitude (41.8 %). Several projects reflected a shift from an initially judgmental attitude to an empathic view of “difficult” patients and families. In about half as many projects, there was a shift from a neutral emotional position at the start to either a positive (20 %) *or* a negative (16.5 %) emotion by the end. Some projects (13.5 %) shifted from negative to neutral. A handful showed a shift from positive to negative emotions (6.5 %). Male students were more likely than female students to describe shifts from a neutral to a positive state. There were more shifts in Group 1 (40.6 %) vs. Groups 2(30.0 %) and 3 (29.4 %). There were fewer neutral to positive shifts in Group 1 than Groups 2 and 3 (17.4 %/23.5 %/20.0 %); more neutral to negative shifts in Group 1 vs. Groups 2 and 3 (23.0 %/13.7 %/20.0 %); and slightly fewer negative to positive shifts in Group 1 vs. Groups 2 and 3 (36.2 %/47.1 %/44.0 %). 96.9 % of education projects and 88.5 % of art projects had no shifts.. Prose projects had more positive shifts than did poetry (29.2 %/18.7 %), while poetry and prose had approximately equal shifts in a negative direction (9.3 %/7.0 %).

### Gender differences

We discovered few differences between male and female students. Both males and females wrote most often from their own perspectives (55.8 % of males, 48.8 % of females), and next most often from the patient’s perspective (26.0 % of males; 20.2 % of females). Females were more likely to adopt the family member’s point of view than males (7.7 % vs. 2.9 %). Regarding theme, males tended to write about patient negative hospital experiences more often than did females (13.0 %/9.1 %). More females than males created projects focusing on child abuse (5.9 %/3.6 %). There were no gender differences in the types of projects that students chose to complete. Males compared to females expressed more frustration (8.1 %/4.8 %), and more contentment/complacency (5.2 %/1.7 %). When recording patient emotions females compared to males noted more sadness (10.8 %/6.2 %), while males noticed more relief (6.2 %/2.1 %).

## Discussion

The aims of this study were to describe the types of students’ creative projects, points of view adopted, and nature of themes examined over a 10 year period on a required third year pediatrics clerkship; and to investigate the emotions expressed in these creative projects overall and in relation to these other dimensions. We discovered that students tended to use written expression most frequently, especially prose, but employed a wide range of creative forms. This finding is consonant with other studies in which students are given an opportunity to work with creative media [41, 42]. Written projects tended to contain more themes and emotions than did art or education projects. Prose projects expressed hope more often than other types of projects, whereas poetry focused on negative relationships more often than other projects. We also learned that students tended to choose the first person voice, most often their own but also that of patients (education projects used a more neutral, objective tone than other types of projects). The use of the first person is indicative of students’ inclination to engage directly with their own experience or “step into the shoes” of the patient (or occasionally family member). Representing the patient’s or parent’s point of view suggests that the student was trying to closely understand the perspective of that person. This interpretation finds support both from a clinical perspective [43] as well as from evidence in the neurosciences that adopting a first person as opposed to a third person perspective indicates greater emotional connection with the other [44]. It is true that, in the first person voice, students were much more likely to write from their own perspective than that of patients. Thus, they remained most comfortable representing their own point of view, rather than entering into that of another.

The most common themes selected by students had to do with patient/relationship-centered medicine, and most others themes related to humanistic concerns, such as maintaining hope and optimism and understanding the patient’s and family’s illness experiences. Education themes were also popular, especially among art projects, even when the project as a whole was not explicitly educational, which might have been a reflection of students’ association of the “physician educator” role with professional identity formation [45].

Regarding the study’s investigation of emotional expression, we found that students were able to recognize a large range of emotions in themselves, patients and, to a lesser extent, family members. The largest number of emotions expressed was negative. However, the emotions students identified most often in themselves were positive ones of empathy, optimism, hope, and friendliness, indicating an empathic and hopeful outlook. While students also expressed a large number of personal negative emotions in their projects, these were rarely directed at patients, but rather reflected their distress at patient and family suffering. Students were more likely to describe their own emotions when the theme was either positive (their relationships with patients), or personal (stress). They tended to be less likely to describe their own emotions in negative situations. However, in these situations they were able to imagine/observe both patient and parent emotions.

Other researchers have noted that students who are not able to examine and come to terms with their own psychological lives find connecting empathically with others to be difficult [46, 47]. It is encouraging that students in our study seemed capable of engaging emotionally, at least in retrospective reflection, especially as other research indicates that students sometimes seem to have difficulty identifying personal emotions at all [24].

Students also seemed well attuned to patient and, to a lesser degree, parent emotions. Other scholars have noted that opportunities to manage strong emotion in a clinical context provide valuable guidance for students and advance self-efficacy [48, 49]. Studies of experienced clinicians suggest that they often ignore, avoid, or minimize the emotional cues of their patients [50–52]. Students seemed less aware of emotions in residents and attending physicians. This could be explained by a lack of attentiveness to physician emotions, or an accurate representation of physicians’ observable behavior, or both.

Patient/relationship-centered themes were associated with a higher than average number of student emotions per project, suggesting that exploring such themes allowed students to express emotional connection. Health education projects were associated with a moderate number of student emotions and a low number of patient and family emotions, indicative of an increased emotional distance when adopting an educational stance.

Overall, across years, projects tended to demonstrate less personal emotional distress, but also less empathy. Instead students appeared to emphasize “friendliness,” arguably a more superficial emotion. The use of the more neutral and objective third person point of view also increased across the three time groups. This may mean that students in later years were working to strike an appropriate balance between emotional detachment and connection; or it might mean that over time students became more self-protective. More education projects in Groups 2 and 3 compared to Group 1 also suggests that over time students may have favored a less emotionally connected position with patients and families.

Shifts were indicative of the “narrative arc” of the project, i.e., how narrative movement was represented in contrast to a cross-sectional representation. Projects were most likely to present shifts in a positive direction. This finding could be because, in fact, most of the shifts *were* positive. It is also possible that students chose not to reflect on shifts that were in a negative direction because of their desire for stories with happy outcomes. There were more shifts and more shifts in a negative direction in the earlier years of the study, which is likely related to the larger number of negative students emotions found in Group 1. Written projects were more likely to contain shifts than were art or education projects, which might have been a function of the nature of the medium (art tends to portray a cross-section in time, whereas narrative is more chronological) or the theme (education is a more straightforward effort than storytelling).

### Limitations

Since this was an exploratory investigation categorizing and analyzing an innovative type of student educational “product” (i.e., creative projects), caution needs to be used in interpreting the results. Importantly, we must remember that how students represent their interactions with and thoughts and feelings about patients and families is not identical with their actual attitudes and behaviors. We also have to take into an account that the patient/family member perspectives represented are only a portrayal of what the student imagined or observed. As well, because all patients involved in this study were children, this may have influenced students’ responses in a more empathic direction. Students’ reactions to adult patients may be more complicated.

Another limitation is that this study involved only a single institution which may restrict the relevance of the findings to other training sites, as might specific characteristics of the clerkship. In other words, the students’ experiences may have been unique to this clerkship, thus the conclusions of this study would not be easily transferrable to other settings. Further, because the projects were a required part of the clerkship year after year students may have shaped their responses in conformity with previous students’ perceptions of what was expected. Similarly, the fact that students were responding to a required assignment reviewed by faculty and shared with classmates may have skewed the findings in favor of emotionally connected self-portrayals, i.e., tending to describe themselves as empathic or friendly, while emphasizing the frustrations and fears of their patients, so as to make their own reactions appear in a positive light. Future quantitative research could build on these preliminary findings through specific hypothesis generation; and by implementing multi-site investigations. Future qualitative research could focus on in-depth analysis of a smaller number of projects to gain more nuanced insights.

### Implications for medical education

The richness of these student-generated creative projects indicates a need for required reflective learning activities during clinical training to provide windows into the subjective experiences of students. The insights offered from this analysis also suggest the importance of supervision during clerkships that safely addresses students’ concern/anxiety, confusion, and emotional distress. The results support the idea that physician/patient relationship and communication are major issues for students in their clinical training, and supervisors should also pay attention to how these can be skillfully cultivated.

## Conclusion

This analysis of 10 years’ worth of required student projects produced during a third year Pediatrics clerkship suggests that students can use artistic media and narrative to insightfully interrogate their clinical experiences. In this study, students were motivated to probe their own emotions, explore the perspectives of patients and parents; and try to develop good relationships, holistic patient care, and strong communication skills. At least as they represented themselves through their creative projects, many of these students chose to adopt emotionally connected, caring attitudes that were patient- [53] and relationship-centered [54], and portrayed stances that suggested “compassionate solidarity” [55] with patients. Pediatrics faculty should recognize the prevalence of this orientation and do what they can to support and maintain it through encouraging ongoing reflective processes; and by providing opportunities for students to discuss issues of emotional connection and detachment within the context of professionalism.

## References

[1] Raszka W, members of COMSEP and the APA Medical Student Education SIG. Curriculum competencies and objectives. Council on Medical Student Education in Pediatrics. 2011. Available from: http://www.comsep.org/educationalresources/currobjectives.cfm. Accessed September 16, 2015.

[2] Gerbase MW, Germond M, Nendaz MR, Vu NV (2009). When the evaluated becomes evaluator: what can we learn from students' experiences during clerkships?. Acad Med.

[3] Lee A, Sharkey A, McGann K, Sumner W 2nd. Mapping students' clinical experiences to pediatric clerkship goals. Am Med Inform Assoc Ann Symp Proc*.* 2006;1003. PMC1839346 17238622

[4] Elnicki DM, Kolarik R, Bardella I (2003). Third-year medical students' perceptions of effective teaching behaviors in a multidisciplinary ambulatory clerkship. Acad Med.

[5] Wald HS, Reis SP (2010). Beyond the margins: reflective writing and development of reflective capacity in medical education. J Gen Intern Med.

[6] Pedersen R (2010). Empathy development in medical education--a critical review. Med Teach.

[7] Plack MM, Driscoll M, Marquez M, Cuppernull L, Maring J, Greenberg L (2007). Assessing reflective writing on a pediatric clerkship by using a modified Bloom's Taxonomy. Ambul Pediatr.

[8] Guillemin M, Gillam L (2015). Emotions, narratives, and ethical mindfulness. Acad Med.

[9] Eichbaum QG (2014). Thinking about thinking and emotion: the metacognitive approach to the medical humanities that integrates the humanities with the basic and clinical sciences. Perm J.

[10] Chen I, Forbes C (2014). Reflective writing and its impact on empathy in medical education: systematic review. J Educ Eval Health Prof.

[11] Kumagai AK (2012). Perspective: acts of interpretation: a philosophical approach to using creative arts in medical education. Acad Med.

[12] Kind T, Everett VR, Ottolini M (2009). Learning to connect: students' reflections on doctor-patient interactions. Patient Educ Couns.

[13] Fischer MA, Haley HL, Saarinen CL, Chretien KC (2011). Comparison of blogged and written reflections in two medicine clerkships. Med Educ.

[14] Rucker L, Shapiro J (2003). Becoming a physician: students' creative projects in a third-year IM clerkship. Acad Med.

[15] Svenberg K, Wahlqvist M, Mattsson B (2007). “A memorable consultation”: writing reflective accounts articulates students' learning in general practice. Scan J Prim Health Care.

[16] Nevalainen MK, Mantyranta T, Pitkala KH (2010). Facing uncertainty as a medical student--a qualitative study of their reflective learning diaries and writings on specific themes during the first clinical year. Patient Educ Couns.

[17] Shapiro J, Kasman D, Shafer A (2006). Words and wards: a model of reflective writing and its uses in medical education. J Med Humanit.

[18] Sharpless J, Baldwin N, Cook R, Kofman A, Morley-Fletcher A, Slotkin R, Wald HS (2015). The becoming: students' reflections on the process of professional identity formation in medical education. Acad Med.

[19] Lamers SMAL, Bolier L, Westerhof GJ, Smit F, Bohlmeijer ET (2012). The impact of emotional well-being on long-term recovery and survival in physical illness: a meta-analysis. J Behav Med.

[20] Madill A, Sullivan P (2010). Medical training as adventure-wonder and adventure-ordeal: a dialogical analysis of affect-laden pedagogy. Soc Sci Med.

[21] Dyche L, Epstein RM (2011). Curiosity and medical education. Med Educ.

[22] Shapiro J (2013). The feeling physician: educating the emotions in medical training. Eur J Person Centered Healthc.

[23] Benbassat J, Baumal R, Chan S, Nirel N (2011). Sources of distress during medical training and clinical practice: Suggestions for reducing their impact. Med Teach.

[24] Karnieli-Miller O, Vu TR, Holtman MC, Clyman SG, Inui TS (2010). Medical students’ professionalism narratives: a window on the informal and hidden curriculum. Acad Med.

[25] Peters S, Young K, McCracken C (2011). What do medical trainees think is so difficult about communicating with patients?. Patient Educ Couns.

[26] Lefroy J, Brosnan C, Creavin S (2011). Some like it hot: medical student views on choosing the emotional level of a simulation. Med Educ.

[27] Zhou Y, Collinson A, Laidlaw A, Humphris G (2013). How Do medical students respond to emotional cues and concerns expressed by simulated patients during OSCE consultations?--a multilevel study. Public Library Sci One.

[28] Forrest DV (2011). Frontline: teaching affect recognition to medical students: evaluation and reflections. J Am Acad Psychoanal Dyn Psychiatr.

[29] Kasman DL, Fryer-Edwards K, Braddock CH (2003). Educating for professionalism: emotional experiences on medical and pediatric inpatient wards. Acad Med.

[30] Pitkala KH, Mantyranta T (2004). Feelings related to first patient experiences in medical school. A qualitative study on students’ personal portfolios. Patient Educ Couns.

[31] Beca IJP, Browne LF, Repetto LP (2007). Medical student–patient relationship: the students’ perspective [in Spanish]. Rev Med Chile..

[32] Monrouxe LV, Rees CE (2012). "It's just a clash of cultures": emotional talk within medical students' narratives of professionalism dilemmas. Adv Health Sci Educ Theory Pract.

[33] Stratton TD, Saunders JA, Elam CL (2008). Changes in medical students’ emotional intelligence: an exploratory study. Teach Learn Med.

[34] Eikeland HL, Ørnes K, Finset A, Pedersen R (2014). The physician's role and empathy - a qualitative study of third year medical students. BMC Med Educ.

[35] Clay AS, Ross E, Chudgar SM, Grochowski CO, Tulsky JA, Shapiro D (2015). The emotions of graduating medical students about prior patient care experiences. Patient Educ Couns.

[36] Shapiro J (2004). Can poetry be data? Potential relationships between poetry and research. Fam Syst Health.

[37] Smith S, Fryer-Edwards K, Diekema DS, Braddock CH (2004). Finding effective strategies for teaching ethics: a comparison trial of two interventions. Acad Med.

[38] Wald HS, Borkan JM, Taylor JS, Anthony D, Reis SP (2012). Fostering and evaluating reflective capacity in medical education: developing the REFLECT rubric for assessing reflective writing. Acad Med.

[39] Charmaz K (2006). Constructing grounded theory: a practical guide through qualitative analysis.

[40] Strauss A, Corbin J (1997). Grounded theory in practice.

[41] Byars LA, Stephens MB, Durning SJ, Denton GD (2015). A curricular addition using art to enhance reflection on professional values. Mil Med.

[42] Ross PT, Lypson ML (2014). Using artistic-narrative to stimulate reflection on physician bias. Teach Learn Med.

[43] Charon R (1986). To render the lives of patients. Lit Med.

[44] Lamm C, Batson CD, Decety J (2007). The neural substrate of human empathy: effects of perspective-taking and cognitive appraisal. J Cognit Neurosci.

[45] McCurdy SA (2012). Willingness to provide behavioral health recommendations: a cross-sectional study of entering medical students. BMC Med Educ.

[46] Meitar D, Karnieli-Miller O, Eidelman S (2009). The impact of senior medical students’ personal difficulties on their communication patterns in breaking bad news. Acad Med.

[47] Hall JA, Roter DL, Blanch DC, Frankel RM (2009). Nonverbal sensitivity in medical students: Implications for clinical interactions. J Gen Intern Med.

[48] Ratanawongsa N, Teherani A, Hauer KE (2005). Third-year medical students' experiences with dying patients during the internal medicine clerkship: a qualitative study of the informal curriculum. Acad Med.

[49] Radley A (2002). Portrayals of suffering: on looking away, looking at, and the comprehension of illness experience. Body Society.

[50] Hall JA (2011). Clinicians’ accuracy in perceiving patients: its relevance for clinical practice and a narrative review of methods and correlates. Patient Educ Couns.

[51] Blanch-Hartigan D (2013). Patient satisfaction with physician errors in detecting and identifying patient emotion cues. Patient Educ Couns.

[52] Fine E, Reid MC, Shengelia R, Adelman RD (2010). Directly observed patient–physician discussions in palliative and end-of-life care: A systematic review of the literature. J Palliat Med.

[53] Stewart M, Brown JB, Weston WW, McWhinney IR, McWilliam CL, Freeman TR (2003). Patient-centered medicine: transforming the clinical method.

[54] Beach MC, Inui T, Relationship-Centered Care Research Network (2006). Relationship-centered care. A constructive reframing. J Gen Intern Med.

[55] Coulehan J (2009). Compassionate solidarity: suffering, poetry, and medicine. Perspect Biol Med.

